# Dogs and Books as Vehicles of Disease

**Published:** 1875-04

**Authors:** 


DOGS AND BOOKS AS VEHICLES OF DISEASE.—A case of scarlet fever has recently happened in England, in which the disease was communicated to two children by a dog. It is believed that the animal, which had been the constant companion of a scarlet fever patient, had had its hair impregnated with contagious matter. This suggests the possibility of dogs, cats, and other household pets, transferring the malady from one house to another, and renders it advisable to keep them out of the way during the prevalence of the fever. Another little considered source of disease may be books in public libraries, particularly volumes which are freely circulated and which cannot be prevented from reaching the hands of patients afflicted with contagious diseases.—*Scientific American.*

